# Simultaneous Aortic Dissection and Pulmonary Embolism: A Therapeutic Dilemma

**DOI:** 10.7759/cureus.12952

**Published:** 2021-01-28

**Authors:** Baikuntha Chaulagai, Deepak Acharya, Sangam Poudel, Pradeep Puri

**Affiliations:** 1 Internal Medicine, Interfaith Medical Center, Brooklyn, USA; 2 Medicine, National Medical College, Birgunj, NPL; 3 Internal Medicine, Geisinger Medical Center, Danville, USA

**Keywords:** pulmonary embolism, aortic dissection

## Abstract

Aortic dissection and pulmonary embolism are medical emergencies that present with a spectrum of symptoms. Most cases of aortic dissection can present with acute chest pain, though some cases may present with other spectra of symptoms. In rare cases, aortic dissection can present simultaneously with pulmonary embolism. We are presenting a case where we saw aortic dissection and pulmonary embolism simultaneously. This case shows the subtle and atypical presentation of simultaneous occurrence of these two highly fatal diseases. To our knowledge, this case has not been published before.

## Introduction

Aortic dissection presenting with acute chest pain is not so uncommon. Stanford type A aortic dissection is more common than type B [1]. The most common risk factors are hypertension and smoking [2,3]. On the other hand, pulmonary embolism is a common diagnosis, the third most common cardiovascular disease after acute coronary syndrome and stroke [4]. Diagnosis of both aortic dissection and pulmonary embolism can be done by CT angiography (CTA) in an emergency setting easily due to its feasibility. It is both sensitive and specific [5-7]. Although aortic dissection usually presents with chest pain, it can also present with other non-specific symptoms [8]. Pulmonary embolism can also present with chest pain or non-specific symptoms, and diagnosis requires good clinical judgment. The clinical criteria should be followed before going for a CT pulmonary angiogram/ventilation-perfusion (CTPA/V-Q) scan as only about 5% of suspected pulmonary embolism are actually diagnosed with pulmonary embolism [9]. On rare occasions, aortic dissection can occur with pulmonary embolism. This presents as a challenging case of a therapeutic dilemma as anticoagulation is indicated in pulmonary embolism and anticoagulation can increase the risk of bleeding in aortic dissection [8,10]. Because of high mortality, early diagnosis and judicial treatment are important to prevent fatalities in both aortic dissection and pulmonary embolism.

## Case presentation

A 61-year-old African American male with a history of hypertension, seizure disorder due to traumatic brain injury since age 30, and alcohol disorder was admitted from the detox unit (day 2 in detox unit) of our institution for nausea, vomiting, and dizziness. Blood pressure was elevated (160/100 on the right arm and 155/98 on the left arm) on admission. The heart rate, temperature, and respiratory rate were normal. Oxygen saturation on room air was more than 95%. Routine blood work CBC (complete blood count), CMP (comprehensive metabolic panel), PT/PTT/INR (prothrombin time/partial thromboplastin time/international normalized ratio) were normal. EKG (electrocardiogram) showed normal sinus rhythm, normal PR interval, and no ST-segment changes. Troponin was negative. The chest X-ray was normal. Urine toxicology was positive for cocaine. Symptomatic management was done for nausea, vomiting, and Procardia® (nifedipine) was given for blood pressure.

**Figure 1:**
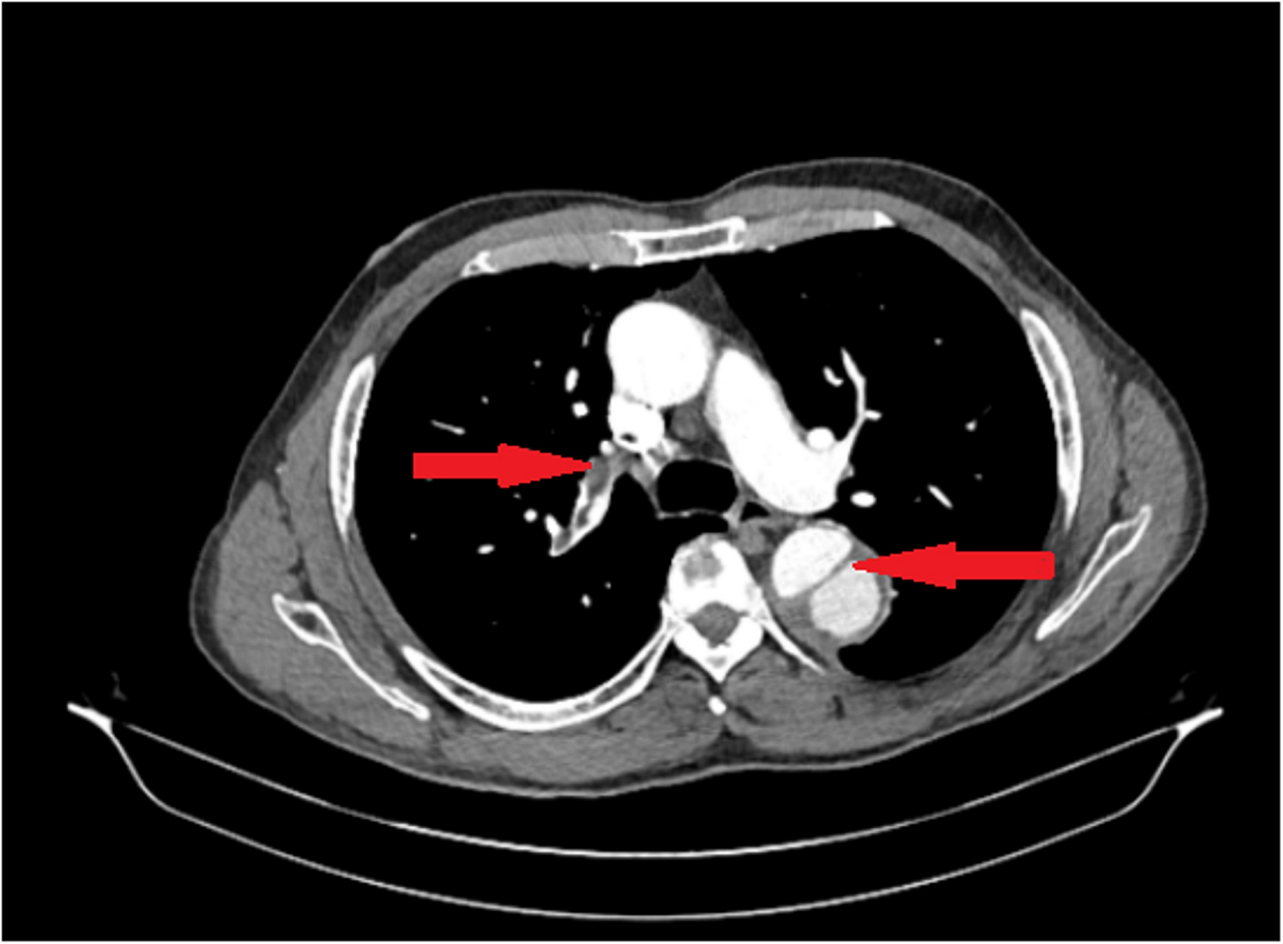
CT angiography showing embolus and aortic dissection in descending aorta

**Figure 2:**
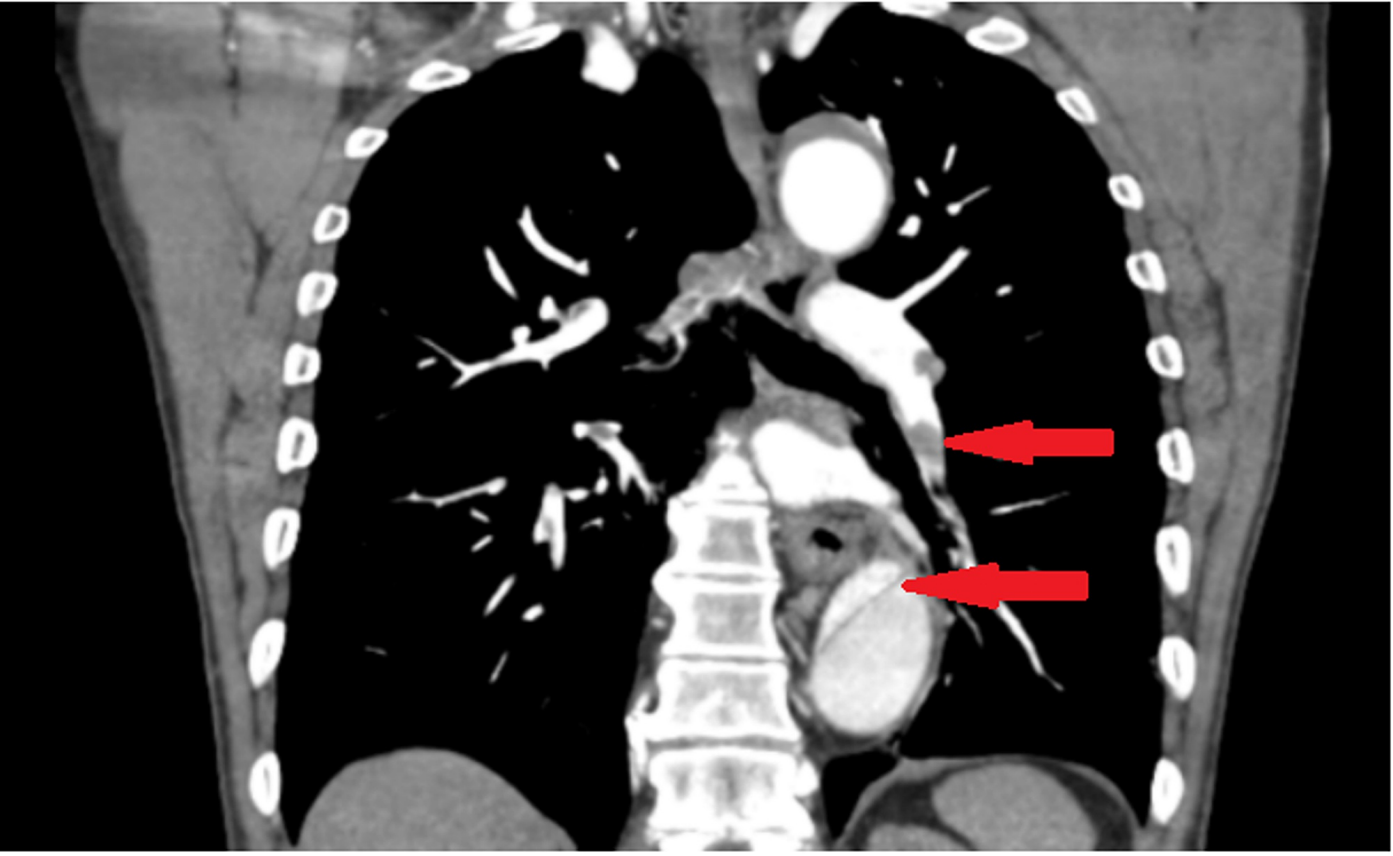
Coronal view showing small emboli and aortic dissection in descending aorta

**Figure 3:**
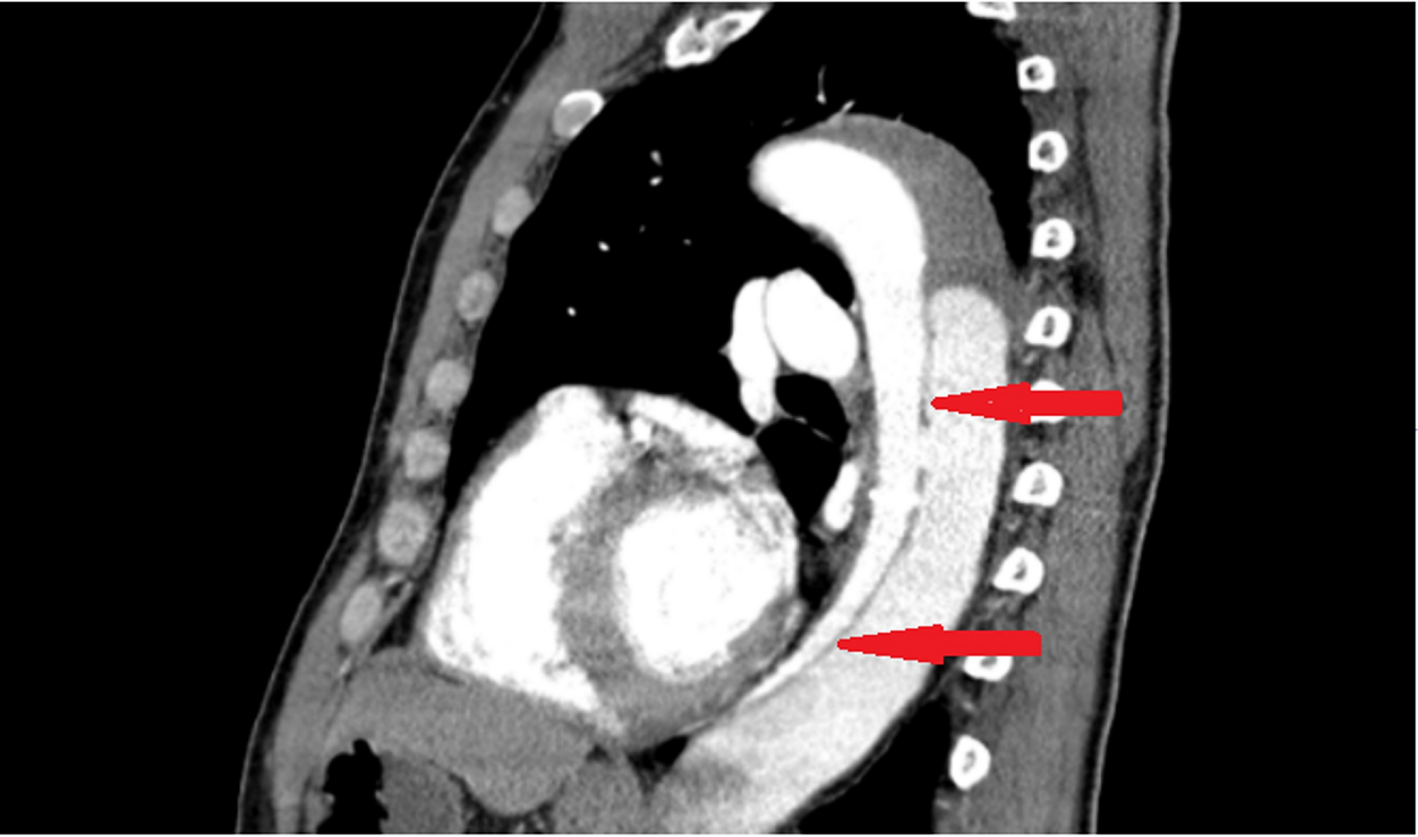
Sagittal view showing aortic dissection in descending aorta

On the medical floor, the patient complained of new left shoulder pain on the same day. EKG and a CT scan of the shoulder were done. CT shoulder showed dislocation of the acromioclavicular joint and sub-solid nodular densities in the left lung. EKG showed no new changes. CT chest was done for sub-solid nodular density seen in the left lung. CT chest showed aortic dissection and CTA was done for confirmation as per the radiologist's recommendation. CTA showed aortic dissection beyond the subclavian artery extending to the level of aortic bifurcation with decreased perfusion to left kidney and bilateral iliac artery aneurysm and multiple bilateral pulmonary embolisms (Figures 1-3).

The patient did not have any history of coagulation disorders or any history of previous pulmonary embolism or deep vein thrombosis. Wells score was 1.5 (low probability for pulmonary embolism). BMI was 24.3 kg/m2. The patient transferred to the coronary care unit (CCU) and the labetalol drip started. The patient did not have any respiratory symptoms and arterial blood gas (ABG) was normal.

On the next day, blood pressure was normal and saturation remained more than 95%. EKG showed no new changes. For pulmonary embolism, the patient was closely monitored. Saturation and ABG remained normal. Anticoagulation was not used due to an increased risk of bleeding. As the dissection was Stanford type B, no surgical intervention was done. Vascular surgery was consulted for a second opinion. Medical management and close monitoring were continued. A venous duplex of lower extremities was done to rule out deep vein thrombosis as the cause of pulmonary embolism; no deep vein thrombosis was seen. An inferior vena cava filter was placed as prophylaxis to prevent further pulmonary embolism. Blood counts and the metabolic panel did not change over the days and vitals remained stable. Blood pressure was controlled with labetalol. Shoulder pain, nausea, and vomiting resolved. No further complications were seen while in the hospital. The patient was discharged with vascular surgery follow up and to complete coagulation workup as an outpatient.

## Discussion

Simultaneous occurrence of aortic dissection and pulmonary embolism is not so common; some cases have been published about their simultaneous occurrence [3,5,11-14]. Though both of the cases can present with atypical chest pain, either aortic dissection or pulmonary embolism is the main presenting complains and the other one is an incidental finding in most cases. In our case, symptoms of aortic dissection were more prominent, and pulmonary embolism was an incidental finding.

Aortic dissection can be a consequence of hypertension secondary to pulmonary embolism, but a pulmonary embolism is rarely a complication of aortic dissection [15]. The most common cause of aortic dissection is hypertension, and it commonly presents with chest pain, though symptoms vary with the severity of the disease [7]. Aortic dissection is usually seen in hypertensive patients who are smokers and have a recurrent history of hypertensive urgencies.

Cocaine use can also cause hypertensive urgency, ultimately leading to aortic dissection, and Stanford type B being more common. Literature shows aortic dissection is associated with cocaine use [16-18]. In our case, the patient was a smoker, and cocaine can be the triggering factor for aortic dissection. 

The incidence of aortic dissection is about 15 cases for 100,000 patient-years and accounts for 85-95% of acute aortic syndromes [3,19]. Pulmonary embolism has an incidence of about 112 cases per 100,000 patient-years [3]. Both of these cases have high mortality and the mortality rate further increases when they present together. The presenting complaints of pulmonary embolism can be chest pain, besides dyspnea, cough, and hemoptysis [9]. Presenting symptoms of pulmonary embolism depend upon the size of the embolus; small sub-segmental pulmonary embolism can be asymptomatic whereas the saddle embolism can present with hemodynamic instability and carries severe mortality unless urgent intervention is taken [9].

As chest pain can be an early warning sign of fatal diseases, the complaint of chest pain should never be ignored. Initial workup for chest pain includes EKG, chest X-ray, and routine blood works. A wide aorta in chest X-ray raises suspicion of aortic dissection. When suspected, diagnosis of aortic dissection can be made by trans-esophageal echocardiography, MRI, and CT angiogram. Both in the cases of aortic dissection and pulmonary embolism, lab values like CBC and CMP are usually normal. D-dimer can be elevated in both aortic dissection and pulmonary embolism [20]. In our case, the D-dimer was not sent as both of the findings were incidental and the presentation was atypical. CT angiogram is the imaging of choice because of its availability in the emergency department and can diagnose aortic dissection as well as pulmonary embolism [5]. As both of these conditions can present with non-specific symptoms to hemodynamic instability based on the severity of the disease, a high degree of clinical suspicion is necessary to diagnose both of these conditions as both carry very high mortality unless intervention is taken [7,9].

Management of aortic dissection depends upon the type. Stanford type A requires surgical intervention while type B can be managed medically [7]. Our case presented with Stanford type B, so no surgical intervention was taken. The mainstay of management of pulmonary embolism is anticoagulation. Thrombolytic therapy might be necessary for massive embolism [9]. But when pulmonary embolism presents with aortic dissection, anticoagulation cannot be given due to risk of recanalization or re-dissection of the thrombosed false lumen and presents as a case of therapeutic dilemma [10]. A judicial decision is necessary to manage the case and might need a referral to a higher center.

## Conclusions

Simultaneous occurrence of pulmonary embolism and aortic dissection is a rare diagnosis. This presents a case of therapeutic dilemma to clinicians. As some of the cases can present with subtle non-specific symptoms, a high degree of suspicion, early diagnosis, and judicial management are very important for mortality prevention. Chest pain should be thoroughly investigated and looked for all possibilities. All other diagnoses should be ruled out before finalizing a treatment because in some cases, treatment intervention of a disease can be detrimental to the other.
